# Our New Normal: Pediatric Nurse Residents’ Experiences with Transition to Practice during the COVID-19 Pandemic

**DOI:** 10.3390/healthcare12121159

**Published:** 2024-06-07

**Authors:** Katherine A. Hinderer, Dennis W. Klima, Marni B. Kellogg, Cecelia Morello, Karen Myers, Beth A. Wentland

**Affiliations:** 1 Institute for Nursing Research and Evidence-Based Practice, Connecticut Children’s, Hartford, CT 06106, USA; bmccollough@connecticutchildrens.org; 2 Department of Pediatrics, University of Connecticut School of Medicine, Farmington, CT 06030, USA; 3 School of Nursing, University of Connecticut, Storrs, CT 06269, USA; 4 Department of Physical Therapy, School of Pharmacy and Health Professions, University of Maryland Eastern Shore, Princess Anne, MD 21853, USA; dwklima@umes.edu; 5 Shriners Children’s, Corporate Headquarters, Tampa, FL 33607, USA; mkellog@shrinenet.org; 6 Departments of Psychology and Public Policy & Law, Trinity College, Hartford, CT 06106, USA; cmorello@trincoll.edu; 7 College of Nursing, University of Phoenix, Phoenix, AZ 85040, USA; kmmyers13@email.phoenix.edu

**Keywords:** pediatric nurse, nurse residency, novice nurse, COVID-19 pandemic, nursing, qualitative, transition to practice, nurse resident

## Abstract

This phenomenological qualitative study examined the lived experience of pediatric nurse residents’ transition to practice during the COVID-19 pandemic. The purposive sample included nine pediatric nurses, participating in a nurse residency program, who entered the nursing profession during the first year of the pandemic. The setting was a free-standing, Magnet-recognized, pediatric academic medical center in the Northeastern U.S. Individual interviews were audio recorded and transcribed. Narratives were analyzed using a hermeneutic phenomenological approach. Five themes emerged from the data: Our New Normal; The Rules Keep Changing; I’m Not Ready for This (transition to practice); The Toll of COVID; and Shattered Family-Centered Care. Sub-themes emerged in The Toll of COVID theme: COVID and the Nursing Care Environment, Emotional Toll of COVID, Burnout: A Universal Truth, and The Pandemic within the Pandemic. The nurse residents’ narratives uncovered the essence of their uncertainty, sorrow, growth, and resilience. Through the eyes of pediatric nurse residents, this study illuminated the experiences of these novices as they entered the nursing profession amid a pandemic.

## 1. Introduction

Registered nurse new graduates, also known as entry-level registered nurses (RNs), are professionals who have graduated from an accredited school of nursing, successfully passed the licensing exam, and are within their first year of clinical practice [1,2]. Academic programs train nurses to become generalist practitioners [1,3]; therefore, in highly specialized areas of nursing such as pediatrics, the first months to years of practice represent foundational milestones where novices formulate their identity as pediatric nurses while cultivating the requisite knowledge and skills that lead to effective clinical judgment [4,5,6]. The transition from student to practicing professional nurse is a critical stage in career development, and support is an essential component of both transition success [7,8,9] and nurse retention [4,10]. Healthcare organizations adopt nurse residency programs or transition-to-practice programs to support new graduate RNs as they evolve from students to proficient clinicians [3,5,11]. The support and structure of nurse residency programs added a critical element of transition success for new graduate nurses entering the workforce during the coronavirus disease 2019 (COVID-19) pandemic [12,13]. While the literature uses the terms novice, new graduate, new nurse, and nurse resident interchangeably, the term “nurse resident(s)” describes program participants of the current study.

### 1.1. COVID-19 and Pediatric Healthcare

When contrasted with adult-focused healthcare organizations, the pandemic manifested differently in pediatric hospitals and healthcare organizations. In the early stages of the pandemic, as opposed to surges of COVID-19-positive patients, especially the critically ill, pediatric facilities experienced a dramatic decrease in admissions and experienced substantial financial losses [14,15]. As a result, layoffs and furloughs were common, with significant personal financial consequences for pediatric nurses [16,17,18]. As the pandemic progressed, pediatric cases of COVID-19 rose [19]. While some children were critically ill, especially those with multisystem inflammatory syndrome in children (MIS-C), the majority of pediatric COVID-19 cases remained less severe in comparison to their adult counterparts [18,20]. The COVID-19 pandemic created unique challenges for pediatric nurses and healthcare teams [19]. Amidst this global crisis, the impact of COVID-19 on the mental health of children emerged. The national and global public health crisis in pediatric mental health added additional complexity and demand to an already depleted healthcare system [19]. Healthcare organizations and health systems struggled to meet the care needs of children as high nursing turnover, coupled with nursing shortages, led to challenging working conditions [21,22,23]. 

### 1.2. COVID-19 Effects on Nurses and Healthcare Providers 

The nature of nursing work historically placed nurses at risk for developing stress, burnout, and post-traumatic stress [24,25,26,27,28]. There were profound physical and psychological effects of working on the front lines and caring for highly infectious patients during the pandemic [29,30,31]. These effects had long-term implications for some healthcare workers [32,33]. In the United States (U.S.), the national and organizational responses to the pandemic led nurses and other healthcare providers to have apprehension and mistrust in healthcare management [34,35]. Nurses and healthcare professionals felt unsupported or uncared for by healthcare organizations as the lack of appropriate personal protective equipment (PPE), poor communication, and constantly changing policies became the norm [13,33,36,37,38]. Nurses risked their health to care for highly infectious COVID-19 patients [39]. Globally, nurses on the front lines at greatest risk of contracting COVID-19 did not receive fair compensation or hazard pay [40]. Many health systems and organizations did not provide paid leave, financial, or other medical-related support for those who contracted the disease and had subsequent sequelae [41,42]. 

Nurses experienced physical exhaustion [41,43], trauma and emotional exhaustion [16,43], isolation from their families [40], and sleep deprivation [30,44] during the pandemic. Front-line nurses described feeling helpless and overwhelmed while being significantly affected by patient deaths attributed to COVID-19 [36]. As a result, over 200,000 experienced RNs left the U.S. nursing workforce between 2020 and 2022 [45]. Psychological effects of the pandemic, including burnout, depression, anxiety, post-traumatic stress, and moral distress, are well documented [16,30,46,47,48]. Occupational burnout was prevalent in nurses and advanced practice nurses working on the front lines [46]. Globally, nurses feared catching COVID-19 and infecting family members with the virus [39,47]. Several studies point to the increased likelihood of poor psychological outcomes and exhaustion among nursing staff, particularly among those who identified as female-gendered [16,29,43]. 

A landmark study conducted in 2021 by the American Nurses Association’s National Commission to Address Racism in Nursing revealed that 63% of 5623 nurses reported experiencing an act of racism in the workplace by fellow nurses, nurse leaders, and patients [49]. Discrimination within healthcare existed before the COVID-19 pandemic. However, whether driven by racial, ethnic, economic, or sexual bias and gender preference, discrimination and racism were accentuated and subsequently worsened during the COVID-19 pandemic [50,51]. Black, Indigenous, and People of Color (BIPOC) nurses faced extraordinary stressors that resulted in increased emotional distress, COVID-19 worry, and burnout [50,52,53,54]. The health equity chasm widened as BIPOC Americans faced a disproportionate number of COVID-19-related deaths [52,55,56]. Longstanding structural and systemic racism [50,51,53,57,58,59], the socio-political environment [60] and the resultant consequences of social unrest, violence, police brutality, and racially motivated murders created an environment fraught with uncertainty, fear, and distress for BIPOC nurses [50,53]. Further, George Floyd’s murder in particular led to increased anger and depression in Black Americans [61]. Misinformation, longstanding racism, and political rhetoric worsened racist acts and discrimination against Asian American Nurses [57,58]. Worsened discrimination increased the COVID-19 pandemic’s negative impact on BIPOC nurses. This led to increased job dissatisfaction, emotional distress, and intent to leave for BIPOC nurses [50,51]. However, the critical need to build and retain a diverse nursing workforce further elevates the importance of mitigating these experiences [50,51]. Of note, Byers and colleagues [53] found that higher levels of psychological resilience ameliorated racism-related stress in nurses. 

Despite these challenges, positive nurse outcomes included post-traumatic growth [16,62,63], compassion satisfaction [64], and feelings of doing good for society [59]. Zhang and colleagues [63] found that social support and self-efficacy were positive predictors of post-traumatic growth in nurses. In contrast, post-traumatic stress disorder severity had an inverse association with post-traumatic growth. 

### 1.3. COVID-19 and Pediatric Nursing

In pediatric nurses, pandemic-era research mirrored concerns expressed by the general nursing workforce. However, the pediatric nurse experience revealed additional considerations related to family-centered care, restrictions in (parent and family) visitation, pediatric nurses’ fear, and difficulty transitioning to care for adult patients with COVID-19 [65,66,67]. The disruption of family-centered care directly resulted from visiting restrictions on parents, siblings, friends, and extended family members [68,69,70,71]. The inability of family units to stay together led to missed child “firsts” [66] and likely affected child well-being. In the neonatal population, the inability to keep the family unit together and the separation of newborns from their mothers and fathers altered breastfeeding and parental bonding [72]. During the pandemic, pediatric nurses in a variety of settings reported or met criteria for anxiety, depression, perceived stress, and psychological distress [73,74]. Burnout and poor quality of life resulted from caring for pediatric patients during the pandemic [67,75,76]. Pediatric nurses and other healthcare workers have described excessive workload due to co-worker illnesses, staffing shortages, patient acuity, and high patient volumes [41,67]. 

Pediatric nurses found comfort in support from colleagues. They expressed positive sentiments around job satisfaction, salary satisfaction, continuous education, and low work violence exposure [67]. These positive experiences correlated with high quality of life and promoted a sense of well-being in pediatric nurses [67]. Pediatric nurses saw their advocacy and support for the patient–family unit as significant nursing contributions. Nurses with higher quality-of-life scores experienced lower burnout during the pandemic [75]. Positive coping supported better mental health outcomes of maternal/child health nurses in China, while social support mediated the stress–health relationship [77]. Teamwork improved colleague and hospital communication, and the perception of meaningful work that contributed to pediatric health. 

### 1.4. Nurses Transitioning to Practice during the Pandemic

Experiences specific to nurses transitioning from student to professional nurse during a pandemic revealed unique challenges and opportunities. Pandemic-related clinical learning restrictions and mandatory stay-at-home laws limited hands-on clinical opportunities. Many schools of nursing had little choice but to employ “virtual” or simulated patient experiences. Research suggests these simulated realities left new nurses feeling unprepared, incompetent, and uniquely challenged with the mastery of clinical skills and communication techniques [38,78]. However, novice nurses acknowledged that educational programs could not have anticipated the complex needs of the pandemic-era practice environment [79].

The transition to practice from student to practicing nurse is stressful. As new graduate nurses embarked on careers as professional nurses during the COVID-19 pandemic, the stress and uncertainty that typically accompany this time in a nurse’s professional career worsened. The stressors and experiences of newer nurses may differ when considering gender identity, sexual orientation, race, ethnicity, and religion, as most U.S. nursing study participants are white females [28,36]. In a study of spring 2020 nurse graduates, Aukerman and colleagues [80] reported that new nurses’ fear related to COVID-19 compounded feeling overwhelmed in their new practice roles. Feeling unprepared and overwhelmed were prevalent themes in studies of new graduate nurses during the pandemic [12,38,78,81]. Moreover, some novices felt unsupported by healthcare team members and described the challenges of working in short-staffed, high-acuity situations [78]. Pandemic-era distancing and isolation practices led to missed opportunities for professional socialization. This further challenged new nurses’ ability to develop relationships with colleagues and build a sense of fit within their profession and team [82]. Professional socialization in nursing supports new nurses’ understanding of the nursing role in the context of unit and organizational culture [82,83].

In addition to emotional and ethical conflicts, secondary traumatic stress, fear of COVID-19, and role uncertainty were prevalent concerns expressed by new nurse graduates [38,80]. During the pandemic, loneliness and isolation were prevalent among new nurses [80]. New graduate nurses demonstrated higher levels of pandemic-era depression than their more seasoned counterparts [84]. Several studies have described the challenges of nurses communicating with their patients due to PPE and wearing a mask, including the limited ability to use facial expressions to support communication [12,80]; this further exacerbated the uneasiness novice nurses felt with communication with patients and families. Some positive experiences realized by new graduates early in the pandemic included feeling appreciated, excited about nursing, and well-supported by co-workers [80]. 

Limited research focused on the experiences of pediatric new graduate nurses or pediatric nurse residents transitioning to practice during the COVID-19 pandemic, especially for those working in free-standing children’s hospitals [38,85]. There is a paucity of research detailing new graduate pediatric nurses’ perspectives on the impact of the pandemic. Using a phenomenological qualitative approach, this study aimed to explore pediatric nurse residents’ experiences as they entered the nursing profession during the COVID-19 pandemic. 

## 2. Materials and Methods

### 2.1. Design and Theoretical Framework

This qualitative study employed individual interviews using the hermeneutic-phenomenological approach guided by van Manen’s methodology [86] to disclose and understand the contextual meaning of the participants emerging into pediatric nursing practice during the COVID-19 pandemic. The philosophical underpinnings of this study are grounded in phenomenology, the study of how individuals understand and endure the human experience [87,88,89]. Phenomenology as a qualitative research method is an approach that guides a researcher to develop a deeper understanding of the human experience as it relates to a particular phenomenon [87,89]. Hermeneutic phenomenology posits that it is through the exploration of the “lived experience” of those who have endured the phenomena of interest that the researcher can begin to understand the meaning of those experiences through description [86,89]. van Manen’s methodological approach to phenomenology is understood in six critical elements: 1. The phenomenon of study is of interest to and adds commitment from the researcher; 2. The experience is studied as it is lived, not conceptually; 3. The phenomenon is characterized with reflection of central themes; 4. The phenomenon is described through writing and re-writing; 5. The researcher maintains strong linkages to the phenomenon; and 6. Research context includes considering parts and the whole [86]. 

### 2.2. Setting, Sampling, and Participant Selection

The nurse residency program at Connecticut Children’s provides support, mentoring, and additional educational opportunities throughout the first 12 months of clinical practice. Residency program participants are newly qualified RNs or novice nurses who previously held a nursing position in a non-pediatric environment for less than 12 months. All new graduate nurses participate in the nurse residency program. The study setting was a Magnet^®^-designated academic, free-standing, non-profit, 182-bed pediatric teaching hospital in the Northeastern U.S. 

After the Institutional Review Board at Connecticut Children’s reviewed and approved this study, participant recruitment occurred via purposive sampling. Participants aged 18 or older who were pediatric nurse residents employed or formerly employed at the institution and within their first year of clinical practice since March 2020 (the onset of the pandemic in the Northeast U.S.) were eligible to participate. In addition, respondents needed to have at least 3 months of pediatric nursing experience at the time of the interview. Nurse residents with previous experience in a non-pediatric setting (new to pediatrics, within the first year of practice) were excluded. Eligible individuals were invited to participate through emails, personal conversations, and announcements during nurse residency classes. Sampling continued until thematic saturation occurred. Interested participants scanned a QR code on flyers or clicked a link in an email if interested in participation. Potential respondents completed an eligibility screen survey housed within REDCap. If eligibility criteria were met, the participants were directed to complete additional intake questions, which included name, the best method to contact, preferred day and time to contact, and preferred day and time for an interview. While the PI knew each of the participants, confidentiality was maintained in several ways. All study materials were housed in secure, locked and/or password-protected locations, either online or in a file cabinet. Only the PI could access the locked files and the original raw data. Each participant was assigned a study ID. Only the PI had access to the list of participant names and study IDs, housed in a separate, secure, password-protected file. All data were de-identified before sharing with the study team.

### 2.3. Data Collection 

Prior to interviews, respondents were provided with the study disclosure and consent form in writing, permission was requested for audio recording and follow-up phone calls for clarification or member checking. The participants chose the date, time, and location of their interviews. On the day of the interview, the researcher contacted the participant to verify availability and willingness to participate. The disclosure and consent were verbally reviewed with the participant at the beginning of the meeting, and any questions or concerns were addressed. Following verbal consent, participants received an email containing an electronic demographic questionnaire and a document that included available resources for support. The investigator confirmed receipt of this information before each interview. Phone interviews were audio recorded. Audio recording was not required to participate in the study; all participants consented to recording. The first author conducted all interviews. A semi-structured interview guide with prompts was available, if needed, to stimulate conversation. The discussion encouraged participants to reflect on their experiences as pediatric nurses and as nurse residents during COVID-19. Silence and clarifying open-ended questions such as “can you describe that experience” balanced the interview and provided an opportunity for participant reflection. Interviews lasted 23 to 50 min. No interviews were halted because of participant distress. Interviews were conducted between January 2021 and August 2021 and were transcribed with NVivo Transcription, an online transcription service.

### 2.4. Team, Rigor, Trustworthiness, and Reflexivity

The research team consisted of several qualitative researchers from nursing (K.A.H., M.B.K., K.M.) and allied health (D.W.K.), experienced pediatric nurses (B.A.W., M.B.K.), and a research student (C.M.). To ensure trustworthiness and integrity, the data were analyzed and collected simultaneously (K.A.H.). Four investigative team members (K.A.H., K.M., B.A.W., C.M.) read and re-read transcripts and kept reflective notes to gain insight into the phenomena of interest. Data were analyzed using reflective writing and team consensus. Direct quotations and continual reference to the data were used as themes emerged. Confirmability was met as two members of the research team, not involved in the data analysis (D.W.K., M.B.K.), reviewed themes and supporting quotations in the final audit trail process. Member checking occurred to ensure the essence of the participants’ experiences was captured. Researcher bias was addressed through multiple independent reviews of the data for thematic analysis and the use of team consensus to support data analysis.

### 2.5. Data Analysis

The phenomenological approach to qualitative data analysis requires reflection on the participant’s experiences to describe and contextualize the phenomenon of interest [87]. During the interviews and after the completion of each interview, field notes documented the researcher’s thoughts and contextual details. A reflective diary organized the researcher’s thoughts during the interview and analysis phases of the study. To capture the experience of the pediatric nurse resident amidst the pandemic, the team read and re-read the nurse resident’s responses multiple times. This led to a deeper understanding of what a new pediatric nurse thought and felt early in their professional nursing career amidst the COVID-19 pandemic. To attain methodological integrity, fidelity to the subject matter through the phenomenological lens was the approach of each interview and subsequent data analyses [90]. We applied an inductive approach to data analysis. Manual coding and NVivo software (Release Version 1.7.1) supported data analysis and coding as themes emerged.

## 3. Results

### 3.1. Participants

Nine pediatric nurse residents participated in this study. All were new graduates in their first positions as RNs. Most were female and white and all were Bachelor’s prepared. Each of the respondents in this study began their nursing careers during the COVID-19 pandemic. Participants represented a variety of clinical settings including ambulatory care areas, critical care areas, and medical-surgical units within a free-standing pediatric hospital. Some completed orientation before vaccines and rapid testing were available and when best practice management for COVID-19 was unknown. Guidance and policies around COVID-19 were constantly changing and fear of the unknown was prevalent. Narratives corroborated the dedication of the nurse resident participants to infants, children, families, and pediatric care. 

**Figure 1:**
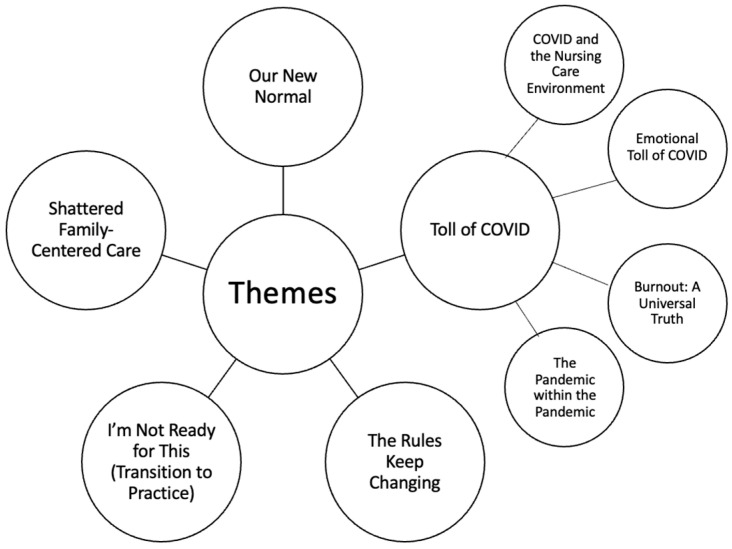
Major themes and subthemes of the study.

Five major themes emerged from the data: Our New Normal; The Rules Keep Changing; I’m Not Ready for This (transition to practice), The Toll of COVID; and Shattered Family-Centered Care (Figure 1). Additional sub-themes emerged in the Toll of COVID theme: COVID and the Nursing Care Environment, Emotional Toll of COVID, Burnout: A Universal Truth, and The Pandemic within the Pandemic (Figure 1).

### 3.2. Our New Normal

The collective experience of nurse residents highlighted the unique environment they encountered at the beginning of their nursing careers. While most worked in health settings (in non-nursing roles) before the onset of the pandemic, their narratives conveyed how they lacked an understanding of the full scope of the pediatric nursing role; moreover, nursing in the COVID-19 era was all they knew. They considered themselves more adaptable to the constant practice changes than their more senior nursing counterparts. For example, one participant shared, “*Maybe I have a different outlook because I have not been in this profession as long*”. Others shared, “…*this* [nursing in COVID-19] *is the new normal; we are trying to figure it out*” as they worked to adjust to the healthcare environment. One nurse stated, “…*if I can handle this, I can get through anything that comes my way*”. The sense of “*doing what needed to be done*” to care for patients underlined their stories of resilience and the emerging new world of nursing amidst the COVID-19 pandemic.

Several of the participants considered their career choices as they watched nurses leave the organization or the bedside. One resident described the loss of an additional level of support as seasoned nurses retired or resigned. They observed turmoil within the pediatric nursing profession. The pediatric nurse residents reflected on comments experienced nurses made such as, “*this is not what pediatric nursing is*” or “*this is not why I became a nurse*”.

One pediatric nurse resident described her excitement with getting her “*number one choice*” unit and the subsequent worry when she realized multiple nurses on her unit were resigning and nurses at other hospitals were furloughed or laid off. Several residents asked themselves, “*Did I make the right choice?*” Connecting with their peers at other regional institutions, some respondents questioned their own personal objectives and workplace stability. For example, one noted, “*We lost staff. I know other hospitals, other places, laid people off*”. Another pediatric nurse resident thought, “*Should I be looking at other hospitals? Is this happening everywhere? It kind of was*”. One nurse shared discussions she had with fellow pediatric nurses, “…*we are wondering, would we still have jobs?*”.

For some, working in COVID-19 solidified their desire to be pediatric nurses. There were feelings of doing good work and being a significant support in the lives of children and families during an especially difficult time. Multiple narratives attested to the positive experience of joining the nursing profession at a time when nursing was well regarded by the public.

*I think we came into the field as nurses at the time when people were finally appreciating how awesome nurses are with the whole pandemic. I feel like there was a huge emphasis on nursing and how nurses are heroes … I think coming into the force when [there was] this really high attitude toward nursing and nursing was a good thing, made me especially feel really excited and proud. To be able to go into nursing during this global pandemic and make a difference. I think that was a really positive thing, coming into nursing at a time when nurses were so needed and so appreciated.*

A new normal emerged related to peer socialization and relationship building that directly contrasted with the pediatric nurse resident’s expectations. COVID-19 prevented a bonding process that happens as new nurses join the culture of their units. One nurse expressed, “*If it weren’t for COVID, I probably would have gone out to dinner with co-workers and bonded with them more. I feel kind of isolated, even making those relationships at work*”. The nurses lamented the missed social opportunities such as Christmas parties and other external gatherings with co-workers. Nurses were unable to eat together in the same room as social distancing prevented breaks and meals with colleagues. The residents commented on not knowing what their peers looked like without masks and the inability to recognize their fellow nurses if they saw them without masks. 

As socialization outside of work was limited during the pandemic, the nurse residency program was a space for nurse residents to socialize and normalize their experiences with their peers. Each shared the importance of the peer connection in the residency as a critical element of their first-year transition. Not all expressed the same level of satisfaction with the residency program; for some, the residency program added to personal stress because of scheduling and implementing an evidence-based project. One shared that the residency “…*added like a large stressor during an already kind of stressful time of being a new grad and dealing with time management and still finding time to do things for myself*”. Comradery and teamwork were critical during this challenging time. One shared, “*we’re all trying to be strong for each other*”. Narratives expressed how COVID-19 brought the nurses and other healthcare team members together. 

### 3.3. The Rules Keep Changing

Participant narratives described concern for pediatric patient care, procedural uncertainty, and worry about the nurse’s safety and health with the constant barrage of new information, policies, and clinical practices. As the pandemic unfolded, the rules around personal protective equipment, patient testing, and patient management procedures frequently changed. Their healthcare role during COVID-19 caused participants to reflect upon their own protection and health consequences. Some nurse residents felt the changes in policies and practices did not always reflect the nurses’ best interests. One participant expressed:

*So I guess my experience of going through it [COVID-19 pandemic] in general, you know, this, it was frustrating. I think the biggest thing was because things were changing all of the time, policies were changing all the time. Best practice is changing all the time, which isn’t usually the case…Whether or not we need to be wearing our N-95 was changing every other day…*

Discussion around the industry’s failure to meet demands for PPE and inconsistency in policy and messaging arose as nurses reflected on PPE requirements. Personal protection was a strong consideration. One nurse asked, “…*am I wearing enough protection? Am I not wearing enough protection?*” Some nurses took personal protection into their own hands. One participant shared a concern about patient and family honesty regarding symptom reporting, “*I can’t keep putting my trust in, you know, that this person [patient and family] says they don’t have a fever…I just was like, whatever, I’m gonna wear my N-95 all the time. And so I think that maybe we all got a little jaded from that perspective … you have to look out for yourself*”. In contrast, one nurse felt very safe in the early days of the pandemic, “*I felt very safe in terms of the PPE that was provided to us*”. Another participant considered the organizational response to the pandemic as a positive experience.

Participants reflected on a time early in the pandemic when so little was understood about pediatric COVID-19 management, “*you’re treating the symptoms and whatnot without really understanding because no one understands what’s going on*”. Nurses expressed a lack of confidence in the treatments for children, especially the medically fragile pediatric population. “*We were fearful, what therapies were going to work for [COVID-19 + and MIS-C] kids?*” COVID-19-related concerns and uncertainties were reflected in statements by residents about surface cleaning, elevator use, and even rules around not eating with each other during breaks because of organizational distancing policies and fear of the spread of COVID-19. 

### 3.4. I’m Not Ready for This (Transition to Practice)

As novice nurses, all participants shared experiences of the transition to independent practice and reflected on the initial lack of confidence and comfort with providing nursing care independently. Several were in orientation at a time in the pandemic when the pediatric hospital census had fallen. They had less opportunity to experience a broader variety of patient scenarios, patient-parent interactions, and pediatric nursing skills. Most participants shared that the transition to off orientation was not what they envisioned. This change, for some, was traumatic. Others experienced being nervous, anxious, or feeling alone. 

One participant called this transition “*drastic*” as they expected to be “*weaned off*” of orientation. Another nurse explained, “*It’s a kind of a whole different ballgame when you’re starting your first job as a nurse, it’s not like you’re doing clinical again… It’s the real deal*”. In an exemplar disclosure, another shared, “*When I first started, it was like, oh my God, so overwhelming. I’ve had those days where you just cry in the bathroom because you’re just so overwhelmed*”. For others, it was not so much the process of coming off orientation but the reality of what independent nursing practice looked like in a pandemic. Several participants described facing new situations and new tasks without having the level of support they thought they would have. Nurse residents faced new challenges as they cared for medically complex children who had experienced trauma, permanent brain injury, or were victims of abuse. Nurse residents encountered unforeseen experiences that included caring for medically complex children who had endured trauma, suffered permanent brain injury, or abuse.

One nurse shared, “*when you get something new and saying, oh shoot, I never got this on orientation and here I am. Everything’s happening no one can really help so I’m kind of on my own*”. Another expressed how on orientation, they were told that as a new nurse, they could step back and use their resources for support (i.e., more experienced nursing staff, unlicensed assistive personnel). However, as the pandemic progressed and patient volumes surged, “*you’re doing everything on your own…it’s really hard when you need someone to help out*”. The narratives illustrated the anxiety and sense of being alone as the pediatric nurse residents transitioned off orientation.

For several nurses, the pandemic forced them to learn time management and organizational skills faster than expected. They quickly learned to care for high-acuity, high-complexity patients. “*The COVID pandemic and stuff has really helped me to develop stronger time management, and maybe a little bit faster than I would have if I wasn’t working in the pandemic*”. Most discussed the importance of their preceptors in the transition to practice. One of the challenges was learning to be on their own without a preceptor right beside them.

As participants reflected on how their practice experience changed over time, some narratives described decreased anxiety and improved comfort in the ability to ask for help. Participants were proud of their personal growth and perseverance over the first year of practice. “*I don’t think I’m nervous as much because I feel more comfortable asking strangers or other nurses that I don’t know on my unit for help. I’m more comfortable with the unknown*”. Reflecting on the end of the first year as a nurse, one resident felt “*proud of growth and independent practice*”.

### 3.5. The Toll of COVID 

The pandemic created an environmental and emotional toll unlike anything these nurse residents had experienced. The practice setting, now riddled with uncertainty, raised concerns about the respondents’ own family members’ health and safety as the pandemic continued. The unfolding behavioral health crisis had significant implications for some areas of nursing practice. COVID-19 affected emotional expression, communication, and socialization, which was unexpected and altered the experiences of these young professionals in unanticipated ways. Many realized that being a new nurse and coming off orientation during COVID-19 was something no other generation of nurses had experienced, which added a layer of stress. Sub-themes in this thematic area included COVID and the Nursing Care Environment, the Emotional Toll of COVID, Burnout: A Universal Truth, and The Pandemic within the Pandemic.

#### 3.5.1. COVID and the Nursing Care Environment 

The pediatric nurse residents reflected on the nature of the COVID-19 pandemic experience in the practice setting. The initial surge of COVID-19 did not affect the pediatric population (and hospitals) as it did in adults. “*As pediatric nurses we were just sitting on the sidelines, the country’s healthcare workforce is getting all this credit, looking at everybody as heroes, and we weren’t*”. Others felt guilt because they were not taking care of COVID-19-positive patients and some even felt lucky that they did not have the same experiences as their adult nursing counterparts. Low patient volumes, canceled cases, and kids staying home began to wear on the nurses’ sense of stability in the future of pediatric nursing. There was great concern over the need to care for adult patients. “*We were talking about if we kept getting furloughed, if we would have to go over to (an adult hospital) and have a partnership with them and work in their E.R.?*” The thought of having to become “adult nurses” was incomprehensible for some. 

Additional practice environment considerations were isolation and resource limitations when caring for COVID-19-positive patients. Narratives spoke to the experience of caring for patients in COVID-19 isolation rooms that amplified the feeling of being alone and unsupported. Due to policy and personal health concerns, early in the pandemic, time spent in COVID isolation rooms and the number of staff entering these rooms were limited. Many shared their first experiences of caring for COVID-19-positive patients as especially difficult.

*I remember, quickly coming off of orientation and being thrown into COVID rooms and I remember it was probably in December. So right when vaccines were just about to come up and I hadn’t worked in many COVID rooms previously. Something about coming out and feeling unclean and dirty and this whole anxiety cloud surrounding that.*

Working with COVID-19-positive patients and being in COVID isolation rooms added a layer of intensity and isolation for these nurse residents. One nurse expressed feeling “*locked in*” and that the isolation added anxiety to an already stressful time in their career. This nurse also explained the challenge of being alone in a room with parents who were upset, “*I remember a parent being very upset with me or just upset in general and yelling and feeling trapped in there*”.

The use of PPE created barriers in communication, expression, socialization, and recognition of others. Several pediatric nurse residents described how much they had to rely on their eyes to share emotions. The masks added more complexity to communication with patients and families, a skill these nurses were trying to master early in their careers. Participants described the importance of choosing words wisely and that masks blocked visual communication cues and expression. One nurse stated, “*I’m new, I don’t really know what to say, my face can’t help me there*”. One nurse described feeling like a monster with blue hands. Another reflected on how critically ill pandemic-era newborns had never seen a face without a mask. This nurse shared, “*they’ve* (the babies) *never seen their own family’s faces, they don’t even know what mouths are because we’re all in masks*”. Wearing PPE made it difficult to hear and understand others; this was especially stressful in emergency situations. 

#### 3.5.2. Emotional Toll of COVID 

Many of the nurse residents described fears around COVID-19, especially early in the pandemic when so much was unknown. There was a fear of contracting COVID-19 or passing COVID-19 to family, friends, and loved ones. Several nurses worried about exposing their parents, who were in higher-risk populations, or their roommates. One nurse shared, “*I was just so petrified because my mom was kind of up there at age and I just didn’t want her getting sick, I just lost my father… the only thing I could think of is my mom*”. Some limited family and social interactions to curb potential familial exposure. One nurse expressed concern for her community as a whole. 

The emotions felt by these participants were unlike what they had previously endured. Narratives included descriptions of anxiety, fear, isolation, and distress. One nurse, speaking about their ongoing anxiety said, “*I never thought it would continue to this point*”. Another shared: “*…the hardest thing for me is actually seeing my co-workers… seeing my co-workers who are relatively strong men and women break down is more hurtful*”. One resident shared, “*it’s been kind of like a roller coaster, that’s the best way to describe it. Like so many different emotions*”. Others conveyed a sense of guilt related to not living up to expectations, “*I don’t feel like I’m providing a good job to some people…because of how busy it’s been, how acute some patients are*”.

The emotional toll of COVID-19, coupled with the added stress of “*seeing violations of health and human rights for other [BIPOC] individuals*”, heightened feelings of vulnerability for new nurses. The BIPOC nursing experience, already fraught with traumatic experiences fueled by racism, worsened during the pandemic. One nurse shared:

*I think one of the worst weeks I feel like in this…[was] the week you where rioting was going on and it was on every single television. And then when I would come into the room and introduce myself as their nurse, you know, people are turning off T.V.s because it’s very awkward and nobody, even staff wise, knew what to say to each other.*

Some found solace in the activities during the residency program, such as journaling and self-care. One pediatric nurse resident told a story of how in nursing school there were self-care days where “*everyone would roll their eyes and be like uh, this again*” recognizing that now, self-care was more important than ever. Both mental and physical health and taking time for self-care were critical. Disregarding self-care affected patient care. One shared, “*It’s like you can’t pour from an empty cup if you just don’t have it to give. And it’s just not safe*”. Another explained, “*It’s really easy to lose sight of the fact that you have to, it’s not being selfish, but you do have to put yourself first because if you’re not caring for yourself, then you can’t care for your patients. If you’re starving you’re not thinking straight*”. The participants acknowledged the challenges of “*taking stuff home*” and the inability, at times, to leave work at work. 

#### 3.5.3. Burnout: A Universal Truth 

From the earliest moments of their nursing careers, these nurse residents noted the prevalence of burnout among pediatric nurses. One nurse saw burnout in nurses in the pandemic as “*a universal truth in different ways, like different shades of the same color*”. The nurse residents reflected on how the pandemic was taxing different generations of nurses in various ways. Some talked about the stresses of nurses’ obligation to their own families and children, while others reflected on how seasoned nurses were deliberating about retirement versus working through the pandemic. Watching the emotional toll of COVID-19 on other nurses, many described feeling compassion and sorrow. The residents observed that more experienced nurses appeared burned out when they (nurse residents) were new and excited to begin their nursing careers. One nurse said, “*Oh my gosh, I’m a new nurse, is this going to happen to me?*”

There were elements of the practice environment that contributed to burnout. As the pandemic progressed, patient acuity rose, and staffing challenges surged. “*We’re just so short-staffed consistently. And that was wearing us down and it’s still wearing us down as a unit*”. Emotional well-being was further complicated by feelings of exhaustion and being overwhelmed. There was a sense of acknowledgment and understanding related to nursing burnout. One nurse talked about the difficulty of finding things to distract from the stress of the pandemic. Another shared that soon after completing orientation she was developing burnout. She explained, “*Sometimes you need to crash and burn to realize what you’re doing isn’t working and I need to change that*”, as she reflected on working too many extra shifts. 

#### 3.5.4. The Pandemic within the Pandemic

For many of these pediatric nurse residents, the behavioral/mental health crisis contributed to the complexity of nursing amid the pandemic. Narratives spoke of the waves of children presenting with mental/behavioral crises and how unprepared they felt to care for this population. The lack of pre-licensure educational preparation related to pediatric mental health was evident in these stories. One resident nurse shared that a healthcare worker from a different discipline, with specialized training in behavioral health told the nurses to, “*just get over it* [the increased behavioral health census]”. The nurse reflected, “*This is the job you signed up for, this isn’t the job I signed up to do*”. Some of the participants shared how they, as younger nurses, were more frequently assigned to mental/behavioral patients than the senior nurses. Others talked about seeing their co-workers break down from caring for the mental/behavioral health population. One narrative explained, “…*if you’ve never been trained in anything like that [pediatric mental/behavioral health] and to be confronted with the, you know, for 14 hours, 13 hours a day and go in and out of that and then come back the next day, and even if you are not sitting for them, you might be their nurse is very traumatizing*”.

### 3.6. Shattered Family-Centered Care

Nurses repeatedly expressed the negative consequences that the pandemic had on family-centered care. Unlike the adult world, pediatrics allowed one parent to remain at the bedside. Siblings, grandparents, and extended family members could not visit and the hospital was no longer a family-centric environment. Visiting restrictions were enforced even on the youngest of patients. One shared, “*one of the most difficult things for me was telling parents only one parent could go upstairs with their sick kid….I had to tell a mother and father of a newborn that only one parent could stay overnight*”. Another expressed how these restrictions limited the ability of the family unit to stay together, especially those who had other children at home. These narratives revealed an element of moral distress in these residents as family-centered care is at the heart of pediatric nursing.

Several nurses who worked with critically ill neonates described the challenges of babies not being able to meet their whole family. One nurse shared, “*there’s one patient who has been in the hospital their entire life, they’re a baby. This patient has never met their siblings, they facetime, but they’ve never touched each other or been in the same room*”. There was a need for a cultural shift in the way nurses communicated with parents and a need to tailor family education that would have normally occurred with mom and dad at the bedside. Some nurses expressed that nursing creativity helped to overcome some of these barriers. 

Limitations on visiting and family-centered care also impacted nursing workload and experience. As fewer parents were able to stay with their children, “*It was just harder. We have more one to one assignments because parents were unable to be there for their kids*”. One nurse described the stress felt by the mother of a child with COVID-19. As care escalated, the mother was not handling the situation well and trying to maintain control over an uncontrollable situation.

In contrast, many respondents saw the importance of the pediatric nurse as an advocate and additional support for the children and their families during the pandemic. Several reflected on how the pediatric nurse’s role transformed during the pandemic as parents, siblings, and family members were unable to visit or had to limit visiting time. These nurse residents reflected on the nurse as a primary caregiver for the children in the absence of parents and families and cherished this as something special. 

## 4. Discussion

The narratives of the nurse resident participants in this study described their realities as emerging pediatric nurses amid a devastating global pandemic. Nurse residents’ lived experiences revealed resilience and flexibility as they navigated the complexities of pandemic-era pediatric healthcare. The pandemic evoked intense emotions. Meeting the demands of the COVID-19-era work environment and constantly changing policies in pediatric healthcare systems disrupted nursing care [14,15,21,23]. The analysis of nurse residents’ experiences during the pandemic revealed five major themes that encapsulated the challenges and adaptations within their experience. 

### 4.1. Our New Normal

The theme Our New Normal captured the nurse residents’ experiences with the COVID-19-era practice environment as their only “normal”. This new generation of nurse residents believed they were more adaptable and resilient than their seasoned colleagues. In Crismon and colleagues’ [17] study, new graduate nurses reported similar sentiments of greater adaptability over experienced nurses during the COVID-19 pandemic. Younger nurses (<39 years), and those with less than two years of experience, perceived the pandemic as less impactful on nursing practice than more experienced nurses [23]. Nurses in the current study reflected on the resignation or retirement of many experienced nurses in their clinical areas. In some cases, these losses caused the nurse residents to question their career and employer choices. One study described this experience as “witnessing the crumbling of the nursing profession” [38]. Several COVID-19-era studies demonstrated nurses questioning their professional career path (nursing) [18,23]. While some of the current study’s participants expressed occupational uncertainty, many nurse residents were proud to be nurses during the pandemic.

Nurse residents in the current study described missed opportunities for professional socialization during their transition to practice. Professional socialization in new nurses supports a novice’s developing nursing identity and fit within their profession, organization, and team [12,91]. In the current study, some nurse residents described the nurse residency program as their only source of pediatric nursing socialization. The residency allowed them to connect with peers who had similar nursing experiences across the organization. Previous research indicated that nurse residency programs improved retention [10,11]. During the pandemic, nurse residencies supported coping with the transition to practice [12,13]. The experiences of the current study’s participants speak to their acceptance of pandemic-era nursing and their self-described adaptability and resilience. 

### 4.2. The Rules Keep Changing

Early in the COVID-19 pandemic, especially before vaccination and rapid testing were widely available, doubt and uncertainty emerged in the second theme, The Rules Keep Changing. This theme underscored the dynamic and inconsistent nature of changing guidelines and protocols amidst the pandemic. Practice-related uncertainty [35] and difficulty managing numerous practice and policy changes were themes that emerged in COVID-19-era nursing studies [36,37,38,78,79,92]. Nurse residents in the current study expressed frustration and concern over the frequent policy changes and requirements around PPE. In a study of more than 700 healthcare workers, nurses held the lowest levels of trust in organizational policies around COVID-19 [93]. Several nurses expressed COVID-19-related fears about adequate personal protection, self-preservation, and taking measures to ensure their safety (i.e., wearing an N-95 mask at all times). Much like the current study, new graduates in a qualitative study expressed concern over PPE. These respondents questioned whether changes to PPE policies were supply-chain issues or evidence-based decisions [79]. For new nurses in Canada, COVID-19-related student experiences in different hospitals and perceptions of organizational protection for nurses were deciding factors in employer choice [43]. Nurse residents in the current study worried if their medically fragile patients with COVID-19 were receiving the right treatment. For these nurses, the overwhelming amount of rapidly changing information compounded personal safety concerns and the fear of contracting COVID-19. Feeling safe and adequately protected were essential to the participants in this study. 

### 4.3. I’m Not Ready for This

I’m Not Ready for This (Transition to Practice) reflected the nurse residents’ profound transition to practice experiences amidst unprecedented circumstances. While it is common for new graduate nurses to lack self-confidence and feel unprepared, the pandemic exacerbated these feelings and worsened new nurses’ emotional burden [12,38,78,80,81]. Some nurse residents in the current study experienced shocking transitions as they were assigned high-acuity patients and COVID-19-positive children immediately after completing orientation. For some, the reality of the transition to practice was not as seamless as they imagined or were led to believe. A similar study of new graduate nurses described how the reality of a nursing career that began during the pandemic differed greatly from what new nurses had envisioned [38]. Multiple high-acuity patients coupled with high census levels meant teams were stressed, resources were limited, and staffing conditions were not ideal. For some participants in the current study, lack of experience with certain clinical skills and specific patient types caused stress and anxiety. The feeling of being “thrown” into intense patient care situations (especially COVID-19-positive patients) without proper training and little to no support was demonstrated in several new graduate nurse studies [12,78,80]. However, other participants in the current study felt very supported by their teams, colleagues, and individuals they could rely on in difficult situations. Despite the challenging conditions these nurse residents faced, many described the critical element of support their preceptors provided. The value and appreciation for preceptors were evident in their narratives. Research demonstrated that new nurses benefited from strong preceptors and unit/team support during the transition to practice [94,95]. 

### 4.4. The Toll of COVID

The Toll of COVID illuminated the multifaceted consequences of the pandemic on nurse residents, including how the nursing care environment related to the emotional toll and pervasive burnout universally experienced in nursing at this time [16,34,75]. The new pediatric nurse resident experience was marred by the pandemic in so many aspects of their personal and professional lives. This collective theme with subthemes represents some of the most emotionally moving stories of this study. The essence of the struggles and the reality of the toll COVID-19 took on these residents was evident in their narratives. 

#### 4.4.1. COVID and the Nursing Care Environment

The nature of the waves of the pandemic, especially the impact on infants, children, and adolescents, altered the nursing care environment in different ways. While many of the stories presented in this study focused on nurse residents’ difficulties, there was also a sense of growth and accomplishment from the experiences these nurse residents shared. Initially, the low patient volumes in the pediatric hospital created angst. These nurse residents worried about job security as furloughs occurred and nursing shifts were canceled. The immense worry and concern over job security and the implications of downsizing staff in pediatric care were expressed by pediatric professionals early in the pandemic [96]. Another reality for the current study’s nurse residents was the potential to pivot to adult nursing. The participants in this study had only worked as RNs in one free-standing pediatric hospital; they were afraid of caring for adult patients in other hospital settings. For some pediatric nurses, the transition from pediatric to adult caused extraordinary stress, fear, and dread [97]. Some of the nurse residents in the current study expressed guilt or feelings of inadequacy over not facing the same initial challenges as their peers in adult health settings. As the pandemic progressed, the nurses in this study described feeling overwhelmed and challenged in new ways as patient acuity rose and staffing challenges persisted. In a 2022 national survey of U.S. nurses, 89% of over 12,000 nurses reported working in understaffed organizations [21]. 

Caring for COVID-19-positive patients was a source of loneliness and isolation. Nurses in the current study described feeling “*trapped*” in an isolation room, and, like a pariah, they were alone and felt ignored. Isolation and loneliness were common consequences of isolation procedures described by novice nurses [80]. PPE created communication barriers and the nurse residents in the current study needed to adapt their communication styles and methods. Dealing with the raw emotions of parents and caregivers who took their frustrations out on these nurses was particularly painful.

#### 4.4.2. Emotional Toll of COVID

Nursing is an emotional experience [98,99]. New nurses confront the intense realities of practice, such as birth, death, suffering, and hope. To “be with” a child and family as they navigate health and illness, a pediatric nurse must confront and learn to manage their own emotions. While the nurse residents in the current study experienced many “typical” new nurse emotions, there was an added layer of difficult emotional experiences precipitated by the COVID-19 pandemic. The participants all described a fear of contracting COVID-19 and spreading it to family and loved ones. Fear was an axiom for most nurses in the pandemic. Many nurses took extra precautions, while some chose to live apart from their families so as not to infect their loved ones, thus altering family dynamics [37,41,92]. For some, being a BIPOC nurse at a time of sociopolitical upheaval paired with increased racism, community suffering, and workplace discrimination resulted in heightened feelings of vulnerability, stress, and fear. The COVID-19 nursing experience for BIPOC nurses was fraught with emotional distress and resulted in high levels of burnout [50,51,52,53,54].

Feeling isolated and alone—in certain practice situations—and in everyday experiences with social distancing took a toll on these nurse residents. Nurse experiences of being lonely and alone and the intense emotions of being separated from family were acknowledged in studies of new graduate nurses [78,80,100]. 

#### 4.4.3. Burnout: A Universal Truth

Nurse residents noted burnout in their nursing colleagues and friends. Some even shared their own experiences with burnout at a very early stage in their nursing careers. Compared with older nurses, younger pediatric nurses may be more apt to develop burnout [75]. The residents in the current study expressed compassion and sorrow for their colleagues’ suffering. Aiken and colleagues [34] recognized that burnout in nursing was not a new phenomenon; however, burnout in nurses and physicians worsened since the onset of the pandemic [35,41]. Failure of the healthcare industry and the nursing profession to adequately address burnout before the pandemic has resulted in significant consequences for the mental health of nurses, nurses leaving the profession, and nurses’ ability to stay in challenging work environments [16,34,75,76]. 

#### 4.4.4. The Pandemic within the Pandemic

In pediatrics, the impact of the pandemic on the mental and behavioral well-being of children was profound [19,101]. The pediatric nurse residents in this study described the challenges of inadequate preparation and resources to provide the best care to children in mental health crises. Additionally, the experience of working alongside nurses and other health professionals who verbally expressed their discomfort and lack of empathy toward children and adolescents with mental health conditions affected the nurse participants’ practice experiences. Akin to burnout in nursing, deficits in systems and supports for pediatric mental health were prevalent before the onset of the pandemic. The COVID-19 pandemic further exposed the significant system failures to adequately meet pediatric mental health needs [101]. Pediatric mental health is an area that requires ongoing, significant attention. The pandemic underscored the failures and shortcomings of the U.S. pediatric mental health system and drew attention to the inadequate resources available to provide adequate care for these children.

#### 4.4.5. Shattered Family-Centered Care

The final theme, Shattered Family-Centered Care, underscored the disruption to traditional patient-and-family care paradigms, a hallmark of pediatrics and pediatric nursing. This had a profound, distressing effect on pediatric care providers and nurses caring for children in hospital settings [66]. Nurse residents in the current study reflected on the negative consequences of visitation restrictions on their patients and their families. Examples included an inability for parents to share moments as a family with a newborn child, siblings hindered from visiting one another, and limited visitation due to personal or household COVID-19-positive status. Similarly, Aukerman and colleagues [80] described new nurses’ concerns and distress over patients being alone without loved ones and family. The nurse residents in this study were distressed over family disruption. In the absence of parents and family, some nurses cherished the special relationship they had with their patients.

### 4.5. Limitations

There are several limitations in this study. Conducted in one organization, this study may reflect regional and organizational culture and influence on the nursing experience. Nurse residents’ experiences may differ significantly in other organizations and areas of the U.S. or globally. Participants knew the interviewer. To offset bias, we employed a team approach to data analysis and several different methods to verify study findings. The entire research team bracketed personal biases and trustworthiness was ensured through member checking, team thematic code consensus, and an audit trail. Interviews were conducted by phone due to the limitations of the particular point in time in the pandemic. Face-to-face interviews or secure virtual interviews would have added elements of observation to data analyses. While not a limitation, it is the nature of qualitative research to provide rich, thick descriptions of the life world of study participants. Thus, qualitative studies are, by nature, not intended to produce generalizable results. 

## 5. Conclusions

The pediatric nursing profession has entered a new era and a new normal. The narratives shared in this study provide some insight into the meaning of being a nurse resident in the early period of the COVID-19 pandemic. The reader can begin to appreciate the incredible journey these new pediatric nurses endured through their descriptions of raw emotions, resilience, and growth. The disruption of family-centered care, the pediatric mental health crisis, and the moral and ethical challenges faced by COVID-19-era nurses cannot be dismissed. The experiences and outcomes of this generation of COVID-19-era novice nurses may help offset challenges for new nurses in the future, especially those emerging during a health crisis. For healthcare providers in many disciplines, including nursing, medicine, and rehabilitation, the effects of the COVID-19 pandemic continue to impact clinicians personally and professionally. Given the experiences encountered, nurses’ perspectives can serve as a conduit for staff development and clinical practice change. Lessons learned from the impact of a novel pandemic on new pediatric nurses are essential considerations linked to the future of the nursing profession.

## Data Availability

The datasets presented in this article are not readily available because the data are part of an ongoing study. Requests to access the datasets should be directed to the corresponding author.
